# GABARAPL1 acts as a potential marker and promotes tumor proliferation and metastasis in triple negative breast cancer

**DOI:** 10.18632/oncotarget.20159

**Published:** 2017-08-10

**Authors:** Li Ran, Tao Hong, Xinhua Xiao, Liming Xie, Junlin Zhou, Gebo Wen

**Affiliations:** ^1^ Department of Endocrine, The First Affiliated Hospital, University of South China, Hengyang, China; ^2^ Center for Gastric Cancer Research of Human Province, The First Affiliated Hospital, University of South China, Hengyang, China

**Keywords:** GABARAPL1, tumor marker, triple negative breast cancer, proliferation, invasion

## Abstract

GABA_A_-receptor-associated protein like-1 (GABARAPL1) is involved in a variety of cancers. The purpose of this study was to investigate the expression, prognostic roles and functions of GABARAPL1 in triple negative breast cancer (TNBC). Quantitative real-time PCR (qRT-PCR) showed that GABARAPL1 was up regulated in both TNBC cell lines and clinical TNBC tissues. High GABARAPL1 expression level was associated with shorter overall survival (OS) and disease free survival (DFS). Furthermore, inhibition of GABARAPL1 suppressed cell proliferation, tumorigenesis, invasion and metastasis, and induced cell apoptosis. We found that metadherin (MTDH) was a downstream target of GABARAPL1. Inhibition of GABARAPL1 suppressed the mRNA and protein expression of MTDH, and overexpression of MTDH could reverse the effects of GABARAPL1 inhibition, which meant GABARAPL1 performed its function partly through MTDH. Our findings demonstrate that GABARAPL1 acts as a tumor promoter in TNBC partly through MTDH. Targeting at GABARAPL1 could be a potential therapeutic strategy for TNBC.

## INTRODUCTION

Breast cancer is the most common cancer in women worldwide. It is estimated that there will be 249,260 new cases and 40,890 deaths in the United States in 2016 [1]. Outstanding advances have been made in breast cancer management in the past few decades, leading to significant declines in breast cancer deaths. However, the prognosis for most patients with triple negative breast cancer (TNBC) remains poor [2]. Lack of specific marker, there is still no effective target for treatment when it comes to TNBC. Therefore, it is essential to develop a novel therapeutic strategy for more effective treatment of TNBC.

GABA_A_-receptor-associated protein like-1 (GABARAPL1) was originally cloned as an estrogen-regulated message [3]. Chakrama et al. [4] first reported that GABARAPL1 participated in the process of autophagy. Xie et al. [5] found that GABARAPL1 had a repressive role in autophagy in prostate cancer. Du et al.[6] reported that miR-143 inhibited autophagy via , Hengyang, Chinatargeting GABARAPL1 in gastric cancer. And Zhang et al. [7] reported that GABARAPL1 was a tumor repressor and inhibited Wnt signaling through the autophagy pathway. In breast cancer the expression of GABARAPL1 was associated with DNA methylation and histone deacetylation [8]. And GABARAPL1 is required for maintaining normal autophagic flux in breast cancer [9].

The prognostic value of GABARAPL1 in cancers is still not clear. In head and neck squamous cell carcinoma high expression level of GABARAPL1 was associated with poor outcome of patients [10]. But in some cancers, high expression level of GABARAPL1 was related with better outcomes, such as hepatocellular carcinoma [11] and lymph node-positive breast cancer [12]. However, the functions and roles of GABARAPL1 in TNBC are still not clear.

Metadherin (MTDH), also known as astrocyte elevated gene 1 (AEG-1), is frequently up regulated and generally correlated with poor outcomes in cancers, such as colorectal cancer [13], liver cancer [14], lung cancer [15] and glioma [16]. Tokunaga E et al reported that overexpression of MTDH is associated with aggressive phenotype and poor prognosis in invasive breast cancer[17]. Li X et al found that MTDH promotes metastasis via induction of epithelial–mesenchymal transition (EMT) in breast cancer [18]. We’ve previously found that MTDH was up regulated in breast cancer and overexpression of MTDH promoted TNBC cell growth and metastasis. And patients with high MTDH expression level exhibited shorter overall survival (OS) [19]. All these results indicate that MTDH is an important potential target in TNBC. But the correlation of GABARAPL1 and MTDH in TNBC has not been reported yet.

Here, we investigate the expression, prognostic roles and functions of GABARAPL1 in TNBC and explore the association of GABARAPL1 and MTDH in TNBC.

## RESULTS

### GABARAPL1 was up regulated and correlated with poor clinical outcomes in TNBC

**Figure 1:**
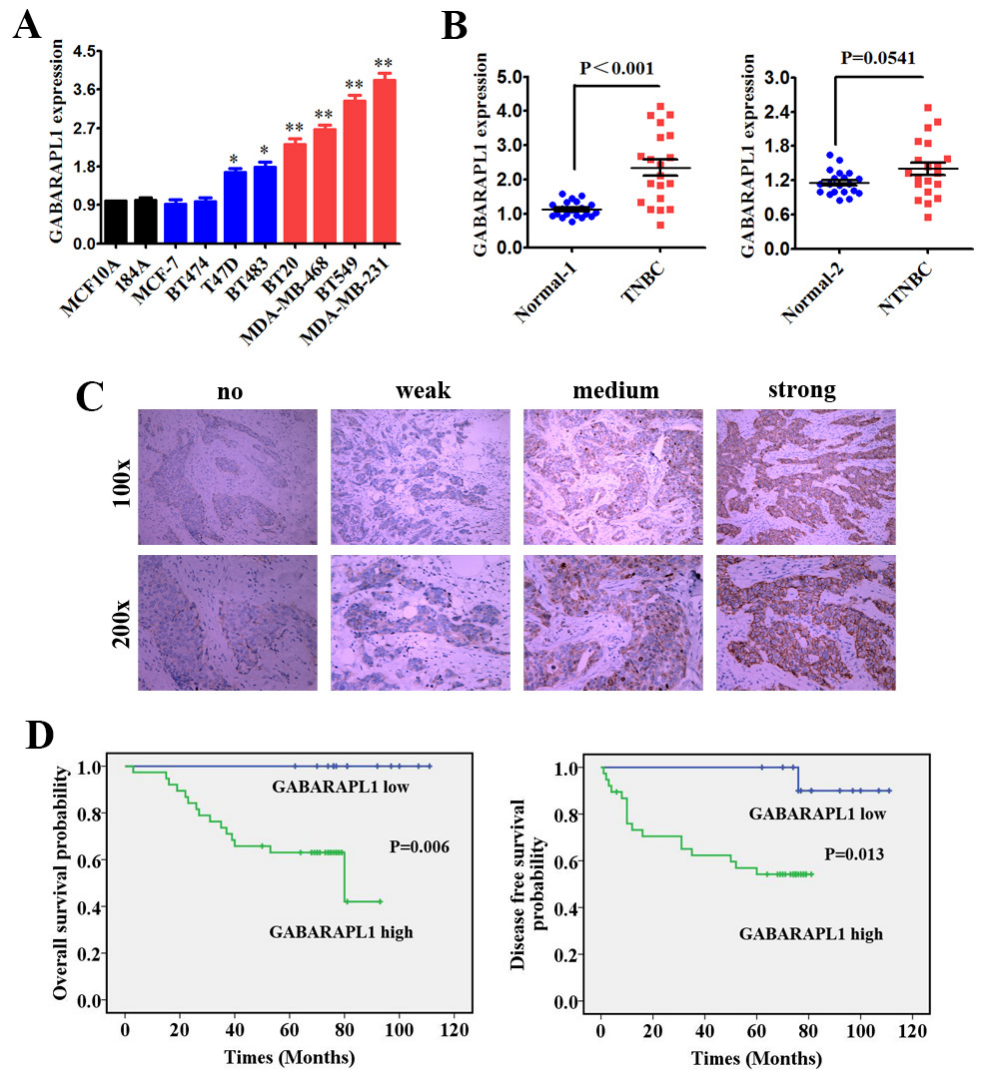
GABARAPL1 was up regulated and correlated with poor clinical outcomes in TNBC **(A)** Expression level of GABARAPL1 was determined by qRT-PCR in two mammary normal cell lines, four NTNBC cell lines and four TNBC cell lines. β-actin was used as an internal control. *P < 0.05, **P < 0.01 **(B)** Expression levels of GABARAPL1 in 20 TNBC tissues and their corresponding paired normal adjacent tissues (Normal-1), together with 20 NTNBC tissues and their corresponding paired normal adjacent tissues (Normal-2). **(C)** Represent pictures of four staining degrees of GABARAPL1 (no-weak-medium-strong) were showed. **(D)** OS (left) and DFS (right) curves for 51 studied TNBC patients with high or low level of GABARAPL1 expression.

The expression level of GABARAPL1 was detected in different subtypes of mammary cell lines by quantitative real-time PCR (qRT-PCR). We found that GABARAPL1 was highly expressed in breast cancer cell lines, particularly in TNBC cell lines (Figure 1A). We then detected the expression level of GABARAPL1 in 20 TNBC tissues and their matched adjacent normal tissues (Normal-1), together with 20 non-triple-negative breast cancer (NTNBC) tissues and their matched adjacent normal tissues (Normal-2). Among the 20 TNBC tissues, approximately 75% (15/20) of the tissues showed notable increase in GABARAPL1 expression level (*P* < 0.001, Figure 1B). However, the GABARAPL1 expression level was only slightly increased in NTNBC tissues (*P* = 0.0541, Figure 1B). These data indicated that GABARAPL1 was up regulated mainly in TNBC.

To further determine the significance of GABARAPL1 in clinical prognosis of TNBC, we performed Immunohistochemistry (IHC) to evaluate the GABARAPL1 expression level in 51 TNBC tissues. The tissues were divided into low or high expression groups based on GABARAPL1 expression level. Represent pictures of four staining degrees of GABARAPL1 (no-weak-medium-strong) were showed in Figure 1C. About 75% (38/51) of patients had high expression of GABARAPL1. Then Kaplan-Meier survival analysis was performed using the patients’ overall survival (OS) and disease-free survival (DFS). The results indicated that patients with high GABARAPL1 expression level exhibited shorter OS (*P*=0.006) and DFS (*P*=0.013) than patients with low GABARAPL1 expression level (Figure 1D). These results indicated that high expression of GABARAPL1 was significantly associated with poor clinical outcomes of TNBC.

### Inhibition of GABARAPL1 suppressed cell proliferation and invasion, induced cell apoptosis in TNBC

**Figure 2:**
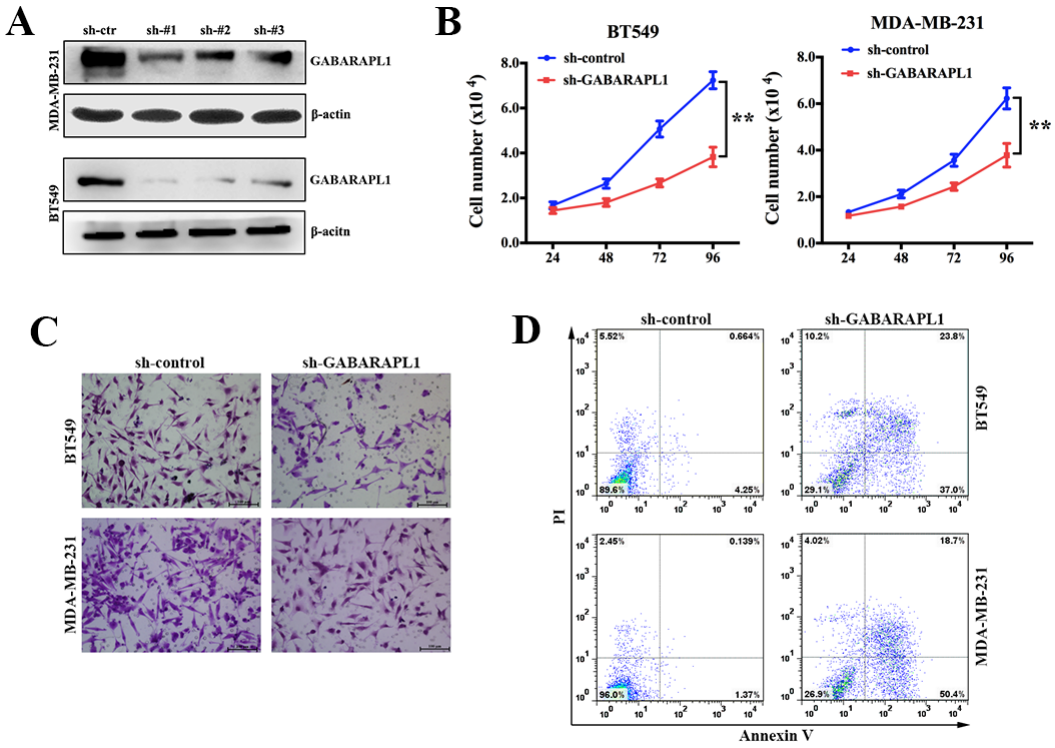
Inhibition of GABARAPL1 suppressed cell proliferation and invasion, induced cell apoptosis in TNBC **(A)** Knockdown effect of sh-GABARAPL1 lentivirus on cell lines was checked by western blot. Sh-#1 was used for further experiments. **(B)** BT549 and MDA-MB-231 cells were infected with sh-GABARAPL1 or sh-control lentivirus. Cell number was counted 24, 48, 72, 96 hours after the infection. **P < 0.01. **(C)** BT549 and MDA-MB-231 cells were infected as above. Then transwell assays were performed to measure cell ability of invasion. **(D)** BT549 and MDA-MB-231 cells were infected as above. Then apoptosis assays were performed.

Noting the inverse correlation between GABARAPL1 expression level and clinical outcomes, we investigated the role of GABARAPL1 in proliferation, invasion and apoptosis of TNBC cell lines. Two TNBC cell lines (BT549 and MDA-MB-231) with relatively high expression level of GABARAPL1 (Figure 1A) were infected with either sh-GABARAPL1 or sh-control lentivirus. Evidence of knockdown is shown in Figure 2A. We chose sh-#1 for further experiments. Then we investigated how GABARAPL1 acted in TNBC cell proliferation. When we knocked down GABARAPL1 in BT549 and MDA-MB-231 cells, we found the cell numbers decreased compared to control group (Figure 2B). Next, transwell assay was performed. As expected, inhibition of GABARAPL1 significantly suppressed cell invasion ability (Figure 2C). Furthermore, we performed apoptosis assay to explore whether inhibition of GABARAPL1 induced cell apoptosis in TNBC cells. Consistent with our expectation, cells infected with sh-GABARAPL1 had an obviously higher ratio of apoptotic cells compared with the control group (Figure 2D).

### Inhibition of GABARAPL1 suppressed tumorigenesis and metastasis in xenograft model

**Figure 3:**
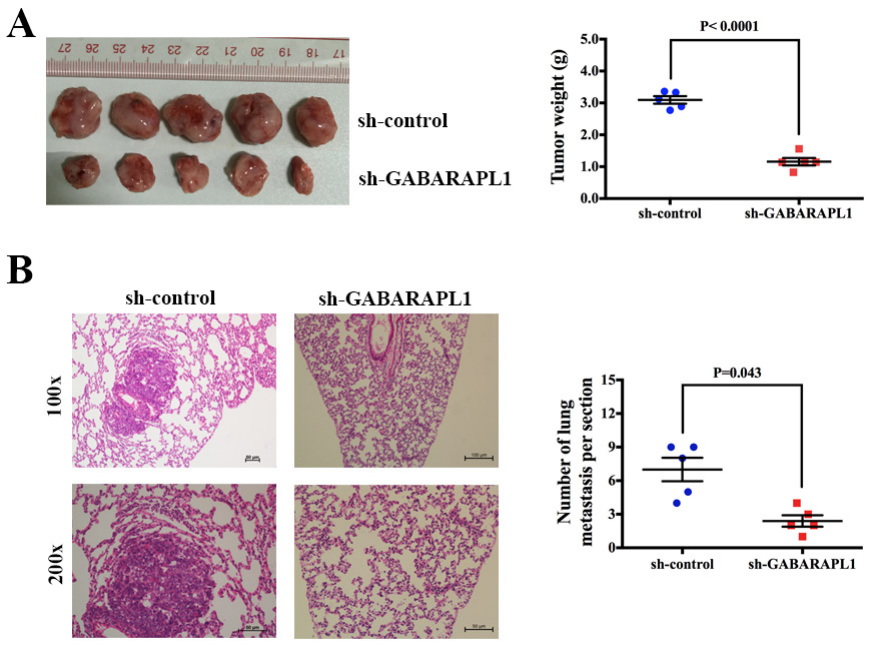
Inhibition of GABARAPL1 suppressed tumorigenesis and metastasis in xenograft model **(A)** Tumor growth in mouse xenograft models. MDA-MB-231 cells infected with sh-GABARAPL1 or sh-control lentivirus were injected to the mammary fat pad of nude mice (five in each group). After 28 days, the mice were sacrificed, necropsies were performed, and the tumors were weighed. **(B)** Tumor metastasis in mouse xenograft models. MDA-MB-231 cells infected as above were injected into the tail vein of nude mice (five in each group). After 28 days, the mice were sacrificed. Micrometastases in the lung per HE-stained section from individual mice were calculated.

To directly evaluate the role of GABARAPL1 in tumor formation and growth in vivo, the xenograft model was adopted. Briefly, MDA-MB-231 cells infected with sh-GABARAPL1 or sh-control lentivirus were injected to the mammary fat pad of nude mice (five in each group). After 28 days, all the mice were sacrificed to harvest the xenograft tumors. We found that the volume and weight of the tumors generated from the sh-GABARAPL1 group was significantly lower compared with the sh-control group (Figures 3A). We then studied the effect of GABARAPL1 on tumor metastasis in vivo. MDA-MB-231 cells infected with sh-GABARAPL1 or sh-control lentivirus were transplanted into the nude mice via tail vein injection (five in each group). After 28 days, the mice were anesthetized, and their lungs were dissected. Haematoxylin and eosin staining was performed to evaluate the tissue morphology (Figure 3B). As shown Figure 3B, a significantly lower number of macroscopic lung metastases could be observed in sh-GABARAPL1 group than in sh-control group. These results indicated that inhibition of GABARAPL1 repressed TNBC tumorigenesis and metastasis.

### MTDH was a target gene of GABARAPL1 in TNBC

**Figure 4:**
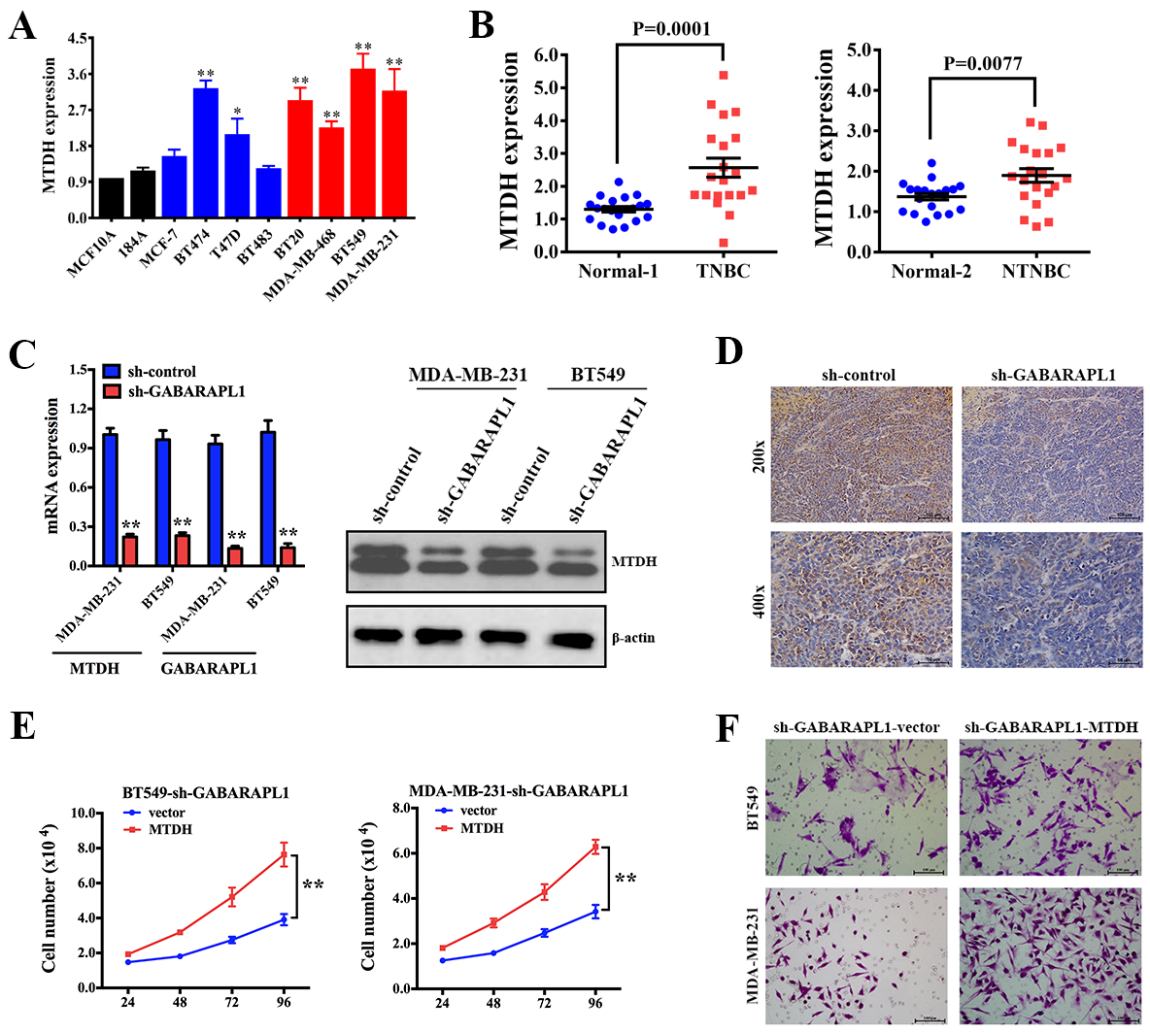
MTDH was a target gene of GABARAPL1 in TNBC **(A)** Expression level of MTDH was determined by qRT-PCR in the above cell lines. β-actin was used as an internal control. *P < 0.05, **P < 0.01. **(B)** Expression level of MTDH was determined by qRT-PCR in the above patients’ tissues. **(C)** MDA-MB-231 and BT549 cells were infected with sh-GABARAPL1 or sh-control lentivirus then qRT-PCR assay and western blot were performed. β-actinwas used as a control.**P < 0.01. **(D)** Representative images of MTDH expression by IHC in the above xenograft tumor tissues. **(E)** BT549-sh-GABARAPL1 and MDA-MB-231-sh-GABARAPL1 cells were infected with MTDH expression vector or control vector then cell number was counted 24, 48, 72, 96 hours after the infection. **P < 0.01. **(F)** BT549-sh-GABARAPL1 and MDA-MB-231-sh-GABARAPL1 cells were infected as above. Then transwell assays were performed to measure cell ability of invasion.

To detect the expression level of MTDH in breast cancer, qRT-PCR was performed in the above cell lines. The result showed that MTDH was upregulated in breast cancer, especially in TNBC cell lines (Figure 4A). We further checked MTDH expression in the above patients’ tissues and found that MTDH was highly expressed in breast cancer tissues (Figure 4B). To explore whether MTDH is a potential target of GABARAPL1, qRT-PCR and western blot analyses were performed. The result showed a notable reduction of mRNA and protein levels of MTDH in cells infected with sh-GABARAPL1 (Figure 4C). IHC staining was applied to detect the expression of MTDH in the above harvested xenograft tumor tissues. The result showed that the expression of MTDH was decreased after inhibition of GABARAPL1 (Figure 4D). To prove that GABARAPL1 functions through MTDH, cell proliferation assay and transwell assay were performed to see whether MTDH could reverse the effects of GABARAPL1 inhibition. And the results showed that after infection of MTDH expression vector, cell number and invasion ability recovered (Figure 4E & 4F). Taken together, these results indicated that MTDH was a downstream target gene of GABARAPL1 in TNBC, that GABARAPL1 functioned partly through MTDH.

## DISCUSSION

Breast cancer is the most common cancer in women worldwide. It is estimated that there were 1,676,600 new cases and 521,900 deaths of breast cancers in women worldwide in 2012 [20]. The last few decades have witnessed the outstanding advances in breast cancer treatment, leading to significant declines in breast cancer death. However, the prognosis for TNBC patients remains poor. TNBC represents 10%-20% of invasive breast cancers and has been associated with worse outcome [21]. Patients with advanced TNBC respond poorly to standard chemotherapy regimens and exhibit rapidly progress. The lack of drug-targetable receptors on TNBC cells has made it difficult to improve the interventions in TNBC [22]. Therefore, it is urgent to develop new specific targeted therapies to better treat TNBC [23].

Recently, GABARAPL1 has been described as a new marker of autophagosomes. And autophagy is becoming a therapeutic target in breast cancer [24]. GABARAPL1 also plays an important role in mitochondrial homeostasis and cellular metabolic programs [25, 26]. The expression of GABARAPL1 is regulated by several factors, such as estrogen [27], androgen receptor [5] and FoxO transcription factor family [28]. In head and neck squamous cell carcinoma, high expression level of GABARAPL1 is associated with poor outcome of patients [10]. But in hepatocellular carcinoma [11] and lymph node-positive breast cancer [12] high expression level of GABARAPL1 is related with better outcomes. However, the functions and roles of GABARAPL1 in TNBC are still not clear.

In our study, we found that GABARAPL1 was up regulated in both TNBC cell lines and clinical TNBC tissues and high expression of GABARAPL1 was associated with shorter OS and DFS (Figure 1). These results suggested that GABARAPL1 could act as a prognostic marker in TNBC. Furthermore, we found that inhibition of GABARAPL1 suppressed cell proliferation and invasion, and induced cell apoptosis *in vitro* (Figure 2). And inhibition of GABARAPL1 suppressed tumorigenesis and metastasis *in vivo* (Figure 3). All these results indicated that GABARAPL1 acted as a tumor promoter in TNBC and it had a tremendous potential as a therapeutic target against TNBC.

MTDH is frequently up regulated and related with worse outcomes in cancers. It is reported that MTDH promotes metastasis via induction of EMT [29]. Wan L et al demonstrated that MTDH plays a critical role in mammary tumorigenesis by regulating oncogene-induced expansion and activities of tumor-initiating cells [30]. Hu G et al revealed that MTDH promotes metastatic seeding and enhances chemoresistance in breast cancer [31]. We’ve previously found that MTDH was up regulated in breast cancer and overexpression of MTDH promoted TNBC cell growth and metastasis. And patients with high MTDH expression level exhibited shorter OS [19]. These findings establish MTDH as an important therapeutic target for breast cancer. In our study, we further confirmed that MTDH was upregulated in breast cancer cell lines and tissues (Figure 4A & 4B). And MTDH is a downstream target of GABARAPL1. Inhibition of GABARAPL1 suppressed the mRNA and protein expression levels of MTDH in TNBC (Figure 4C & 4D). Overexpression of MTDH reversed the effects of GABARAPL1 inhibition, which meant GABARAPL1 functioned partly through MTDH (Figure 4E & 4F).

In summary, this study revealed that GABARAPL1 was up regulated and associated with worse outcome in TNBC. Inhibition of GABARAPL1 suppressed cell proliferation, tumorigenesis, invasion and metastasis, and induced cell apoptosis. MTDH was a downstream target of GABARAPL1, that GABARAPL1 performed its function partly through MTDH. Targeting at GABARAPL1 could be a potential therapeutic strategy for TNBC.

## MATERIALS AND METHODS

### Cell lines and culture

The following cell lines were obtained from the American Type Culture Collection (Manassas, VA, USA) and were passaged in our laboratory for less than six months after thawing frozen aliquots: human breast cancer cell lines (MDA-MB-231, MDA-MB-468, BT549, MCF-7, T47D, BT474, BT20 and BT483) and normal mammary epithelial cell lines (184A1, MCF-10A). All the cells were maintained according to the supplier’s instructions. Before use, all the cell lines were authenticated by short-tandem repeat DNA profiling and were found to be free of mycoplasma infection.

### Clinical samples

Tissue samples of 20 TNBC tissues and their paired adjacent normal mammal tissues (Normal-1), together with 20 NTNBC tissues and their paired adjacent normal tissues (Normal-2) were immediately cut and stored in RNAlater (Ambion) then subjected to qRT-PCR analysis. A total of 51 human TNBC tissues were formalin-fixed and embedded in paraffin by standard methodology then subjected to IHC. Clinical samples were collected between 2006 and 2009 at The First Affiliated Hospital, University of South China. This study was approved by the Ethics Committee and institutional review boards (IRBs) of University of South China Health Authority. The collection and use of tissues followed procedures in accordance with the ethical standards formulated in the Declaration of Helsinki. Informed consents were obtained from all patients included in the study.

### Quantitative RT-PCR analysis (qRT-PCR)

The total RNA from cells or tissues was extracted with TRIzol reagent (Invitrogen, USA). Reverse transcription and qRT-PCR reactions were performed using a qSYBR-green-containing PCR kit (Qiagen, USA). The threshold cycle (CT) value for MTDH and GABARAPL1 were normalized against the CT value for internal control β-actin. The fold change was determined as 2-ΔΔCt. The primers for qRT-PCR detection were synthesised by Invitrogen: MTDH forward, 5’-TGGCAAATGTGGCCAACA-3’; reverse, 5’-TATTAGGTAACCGACCCCCTCTT-3’. GABARAPL1 forward, 5’-CCCTCCCTTGGTTATCATCCA-3’; reverse, 5’-ACTCCCACCCCACAAAATCC-3’. All the qRT-PCR assays were performed with the Bio-Rad IQTM5 Multicolour Real-Time PCR Detection System (USA).

### Immunohistochemistry (IHC) analysis and scoring system

After deparaffinizing and rehydrating, the slides were treated with 90% methanol/3% H2O2 solution for 10 minutes at room temperature to block endogenous peroxidase. Then, the slides were soaked in sodium citrate buffer (10 mM Sodium citrate, 0.05% Tween 20, pH 6.0) under 96°C for 5 min for antigen retrieval. After blocking by BSA, the following antibodies were used: antibody against GABARAPL1 (Santa Cruz, USA) and antibody against MTDH (Santa Cruz, USA). We added antibodies to the slides for overnight storage at 4°C and then incubated the slides at room temperature with biotinylated secondary antibody for 10 minutes, and finally HRP-Streptavidin for 10 minutes. After DAB staining, the results were graded for intensity. The intensities of GABARAPL1 staining were scored between 0 and 4 according to the standards of 0–1 (no staining), 1–2 (weak staining), 2–3 (medium staining), and 3–4 (strong staining). The percentages of GABARAPL1 positive cells in 3 representative high-power fields of individual samples were analysed. Scores of intensity multiplied by the percentages of positive cells equalled to the final scores of GABARAPL1 expression. The maximum was 4 and the minimum was 0. Individual samples were evaluated by at least 2 pathologists in a blinded manner, and those expression scores greater or equal to 2 were defined as high expression, less than 2 was defined as low expression.

### Establishment of stable cell lines with sh-GABARAPL1

Recombinant shRNA lentiviruses containing sh-GABARAPL1 and control shRNA lentiviral particles were purchased from FulenGen (Guangzhou, Guangdong, China). BT549 and MDA-MB-231 cells were infected with sh-GABARAPL1 or sh-control then selected with 5 μg/mL puromycin for two weeks (Invivogen, USA) until drug-resistant cells were obtained. Then these stably transduced cell lines were used in the following experiments.

### Cell proliferation assay

A total of 1×10^4^ BT549 or MDA-MB-231 cells infected with sh-GABARAPL1 or sh-control were plated on 6-well plates. The numbers of cells were counted by trypan blue staining after 24, 48, 72 and 96 hours of incubation using a Coulter Counter (Beckman Coulter, Fullerton, USA) in triplicate.

### Cell invasion assay

BT549 and MDA-MB-231 cells were infected with sh-GABARAPL1 or sh-control then seeded into the upper chamber with Matrigel in the insert of a 24-well culture plate (BD Biosciences, MA) in serum-free medium. Then 15% fetal bovine serum was added to the lower chamber as a chemoattractant. After 48 hours of incubation, Invasive cells adhering to the lower membrane of the inserts were stained with Crystal Violet, counted and imaged. Triplicate independent experiments were performed

### Apoptosis assay

Annexin V/ propidium iodide (PI) staining was performed for the detection of apoptotic cells. BT549 and MDA-MB-231 cells were infected with sh-GABARAPL1 or sh-control then 5×10^5^ cells were collected and washed twice with ice-cold PBS. The cells were then stained using the Alexa Fluor®488 annexin V/Dead Cell Apoptosis Kit (Invitrogen, CA, USA) for flow cytometry analysis according to the manufacturer’s guidelines. The untreated cells served as a negative control. Flow cytometric analysis was performed immediately using a FACSCalibur instrument (Becton Dickinson, USA).

### Mouse xenograft model

A total of 2×10^6^ MDA-MB-231 cells infected with sh-GABARAPL1 or sh-control were inoculated to the mammary fat pad of nude mice (five in each group). After 28 days, the mice were sacrificed, necropsies were performed, and the tumors were weighed. Then tumor tissues were subjected to IHC for detection of MTDH expression. To assay the effect of sh-GABARAPL1 on tumor metastasis, 1×10^5^ MDA-MB-231 cells infected with sh-GABARAPL1 or sh-control were injected into the tail vein of nude mice (five in each group). After 28 days, the mice were sacrificed and necropsies were performed. The numbers of micrometastases in the lung per HE-stained section from individual mice were analysed by morphological observation. All the animal procedures were performed in accordance with institutional guidelines and all possible steps were taken to avoid animal suffering at each stage of the experiment.

### Western blot

Western blot analysis was performed using standard procedures. Briefly, total proteins were extracted and separated by 10% sodium dodecyl sulfate polyacrylamide gel electrophoresis (SDS-PAGE) then transferred onto the PVDF (polyvinylidene difluoride) membrane. Then, the membrane was incubated with antibody against GABARAPL1 (Santa Cruz, USA) or antibody against MTDH (Santa Cruz, USA) followed by HRP-labeled secondary antibody (Santa Cruz, USA) and detected by chemiluminescence. β-actin was used as a protein-loading control.

### Statistical analysis

Comparisons between groups were analysed with t tests. Paired Student's t tests were used to compare mRNA levels between breast cancer tissues and corresponding normal tissues. Kaplan-Meier plots and log-rank tests were used for survival analysis. Unless otherwise indicated, the data are reported as mean ± SD at a significance level of *P* < 0.05. The statistical analyses were performed using the SPSS16.0 software.
